# Supplementary material for: Hemifusomes and Interacting Proteolipid Nanodroplets Mediate Multi-Vesicular Body Formation
Source: Res Sq. 2024 Oct 21:rs.3.rs-5200876. Preprint. [Version 1] doi: 10.21203/rs.3.rs-5200876/v1 (PMC11537336; doi:10.21203/rs.3.rs-5200876/v1)
Supplement: Supplement 1 [file NIHPPRS5200876V1-supplement-1.pdf]

Supplementary Figure Legends

Supplementary Figure 1. Workflow of image acquisition and distribution of hemifusomes and in various cell lines

a. Overview cryo-EM image of the leading edge of a COS7 cell on a cryo-EM grid showing the gradient of ice thickness (inset) and examples of the targets selected for acquisition of “search” images and tilt series for tomography. Scale bar: 10 μm.

b. View of a representative “search” image. Hemifusome labeled HF. Scale bar: 400 nm.

c. Close-up view of a representative “projection” image from a raw tilt series acquisition and corresponding tomographic reconstruction using either the weighted back projection (WBP) or simultaneous algebraic reconstruction technique (SART). Scale bar: 100 nm.

d. Representative search views of HELA 3T3, and COS7 cells with vesicular organelles highlighted in color: endosomes (EN, in pink); multivesicular bodies (MVB, in blue); hemifusomes (HF, in yellow); and flipped hemifusomes (fHF, in green). The COS7 cell image in this panel was used to target and collect the tilt series, and reconstruct the tomogram shown in Figure 1b. Scale bar: 400 nm and 200 nm.

e. Representative, low magnification, overview of a COS7 cell periphery illustrating the frequency of hemifusomes compared to the other vesicular organelles in the cell border. Scale bar: 1 μm.

f. Tomogram slice of a COS7 cell leading edge showing a hemifusome (HF) in a region of cytoplasm with a filopodia and a dense cytoskeletal network. Scale bar: 200 nm.

Supplementary Figure 2. Hemifusome among endolysosomal vesicles in a cultured cell line and in a native epithelial cell

a-e. Cryo-ET images of a COS-7 cell showing organelles at the cell periphery, including: a hemifusome (HF) near a clathrin-coated pit (CP), a clathrin-coated vesicle (CV), and variably sized

endosomes (a); Endosome (En) where a vesicle appears to be pinching off as shown by arrow (b); Lysosome (Ly) with slight membrane invagination decorated with protein particles (c); Lysosome (Ly) with complex content; Lysosome with tubulation depicted by arrow (d).

b. Conventional electron microscopy of a thin section of fixed and plastic-embedded epithelial cell from the frog macula. The image shows the apical cytoplasm of an epithelial cell with a hemifused pair of vesicles among several endosomal vesicles. Scale bars: 100 nm.

Supplementary Figure 3. Morphological variability and deformability of hemifusomes

a-c. Series of cryo-ET mid-cross-sections showing hemifusomes laterally compressed by cytoskeletal elements (a), the plasma membrane (b), or both (c). Asterisks mark vesicles with clear luminal content.

d. Single projection images from a raw tilt series at 0° and at 60° relative to the cryo-EM grid as illustrated in g showing the vertical compression of the hemifusome.

e. Reconstructed tomogram showing slight compression of the vesicle.

f. 3D-rendered segmentation of hemifusome in (e). The golden sphere in represents the proteolipid particle commonly located at the rim of the hemifusion diaphragm.

g. Schematic illustrating the missing wedge effect limitations of cryo-ET, where membranes cannot be visualized above and below a narrow strip of the total vesicle volume. Scale bars: 100 nm

h. Plot of compression factor

i-j. 3D views of segmented volumes of a multivesicular body (i) and a hemifused pair of vesicles (j) illustrating the missing wedge or missing cone effect in the reconstructed tomograms.

Supplementary Figure 4. Range of morphologies of hemifusomes and flipped hemifusomes highlighted in segmented tomograms

a-b. 3D views of segmented hemifusome membranes illustrating various size ratios, roundness, and curvature of the hemifusion diaphragm. These reconstructions are from hemifusomes shown in the main figures.

c. The ratio of diameters of the hemifusion diaphragm (HD) and larger vesicle of the hemifusome (HF). n = 50 hemifusomes.

d. Close-up view of the lens-shaped structure of the smaller vesicle of the hemifusome.
e. 3D views of segmented flipped hemifusome membranes illustrating various degrees of inward budding of the smaller vesicle into the lumen of the larger vesicle.
f. Close-up view of the external hemifusion diaphragm (arrow). Note that some membranes of the flipped hemifusomes show subtle crenation indicative of reduced turgor.

g. Image of direct and flipped hemifusomes to illustrate where measurements were made for membrane bilayer thickness (lines drawn).

h. Graphs depicting pixel intensity along the lines drawn in (g). The maxima in each trace represent each leaflet of the bilayer. Bilayer thicknesses measured: 1= 4.149 nm, 2= 4.105 nm, 3= 4.546 nm, f1= 4.135 , f2= 4.142, f3= 4.55.

Supplementary Figure 5. Pulse-chase of nanogold particles in the endocytic pathways

Tomographic slices examples of pulse-chase experiments showing the uptake of nanogold particles of various surface functionalization and sizes (small particles = 5nm and large particles = 15 nm) in endosomes (En), and lysosomes (Ly), but absent from MVBs and a vesicle hemifused to a lipid droplet. Gold particles are enriched in mature, large lysosomes. T = time of incubation with the gold nanoparticles. TfP = gold nanoparticles with transferrin physiosorbed and NC = gold nanoparticles with slightly negatively charged non-reactive polymer. Scale bars: 200 nm.

Supplementary Figure 6. Proteolipid particles embedded in the membrane of hemifusomes, and flipped hemifusomes

a-b. Tomographic slice representative of additional PNDs embedded in the membrane of hemifusomes (HF) at sites away from the initial HD.

c-d. Tomographic slice representatives of PND at the site of contact of the inner and outer vesicles of a flipped hemifusome (fHF). Asterisks mark vesicles with clear luminal content.

e. Hemifused endosome and lipid droplet (LD). The texture and contrast of the LD is very comparable to that of the PNDs in (a-d), highlighting that the PND very likely is comprised of lipids, but with a proteinaceous coat that contrasts with the lipid monolayer surrounding the LD.

Supplementary Figure 7. Additional examples of compound hemifusomes

a. Panel of cryo-ET slices showing compound hemifusomes and flipped hemifusomes, consistent with the hypothesis that they evolve into complex multivesicular bodies (MVB). Asterisks mark vesicles with clear luminal content.

b - d. Panel of cryo-ET images (top) and corresponding segmentation (bottom) of compound hemifusomes (HF), (b), compound flipped hemifusomes (fHF), (c), and compound fHF with free intraluminal vesicles (d). Often, the compound flipped hemifusome shows a slightly crenated membrane indicative of reduced turgor pressure. Scale bars: 200 nm.

Supplementary Movie 1 and 2. Visualization of direct hemifusomes in COS7 cultured cells.

Tomographic reconstructions of direct hemifusomes in a COS7 cell reveal a dense structure embedded within the hydrophobic interior of the bilayers at the junction of HD and two heterotypic vesicles. Videos are displayed at 24 fps. Scale bar: 100 nm.

Supplementary Movie S3: Visualization of flipped hemifusome in cos7 cultured cells.

Tomographic reconstructions of flipped hemifusome in a COS7 cell show a translucent vesicle hemifused to the luminal or exoplasmic side of the larger vesicle membrane. The videos are displayed at 24 fps. Scale bar: 100 nm.

Supplementary Figure 1

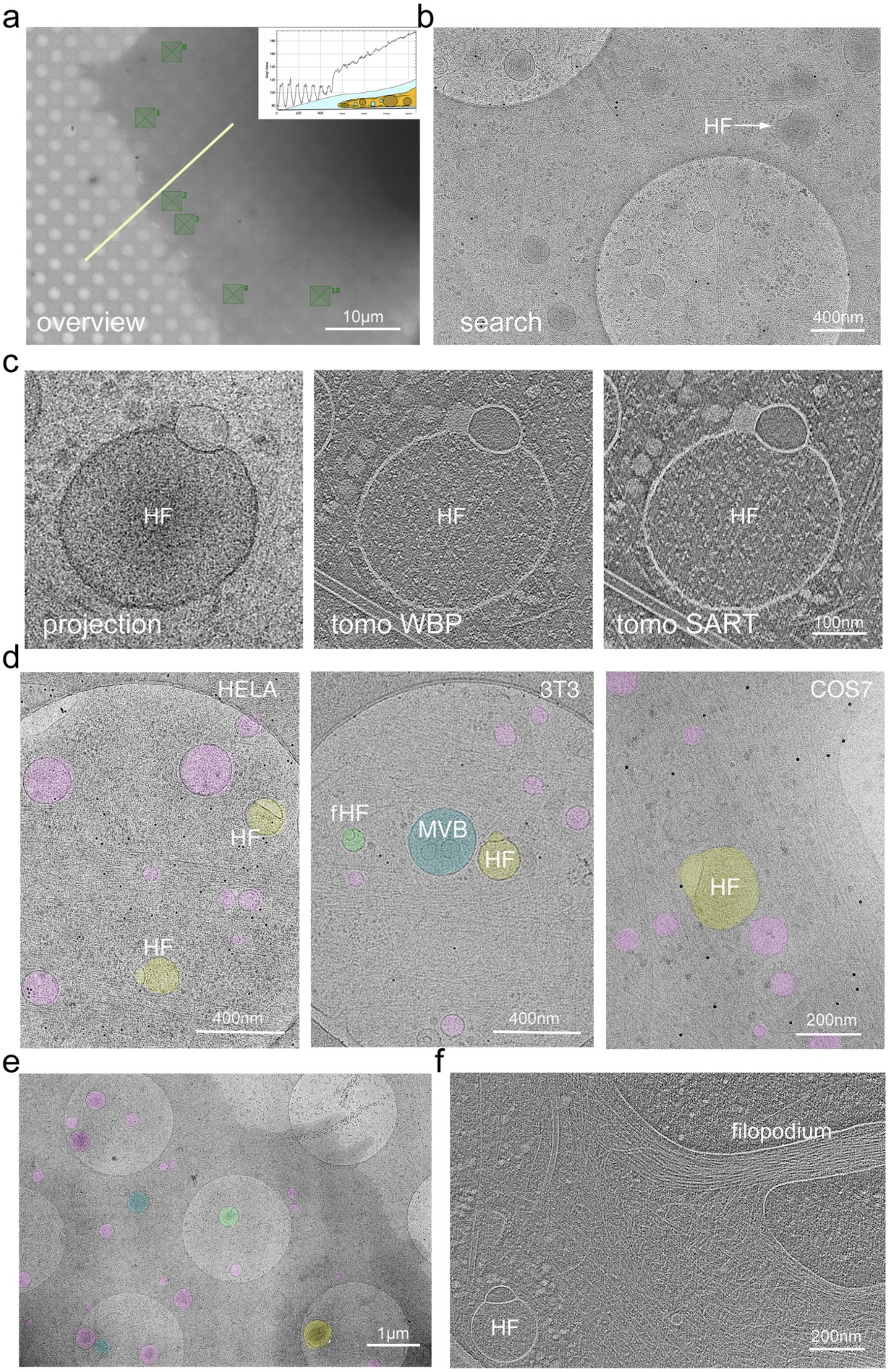

Supplementary Figure 2

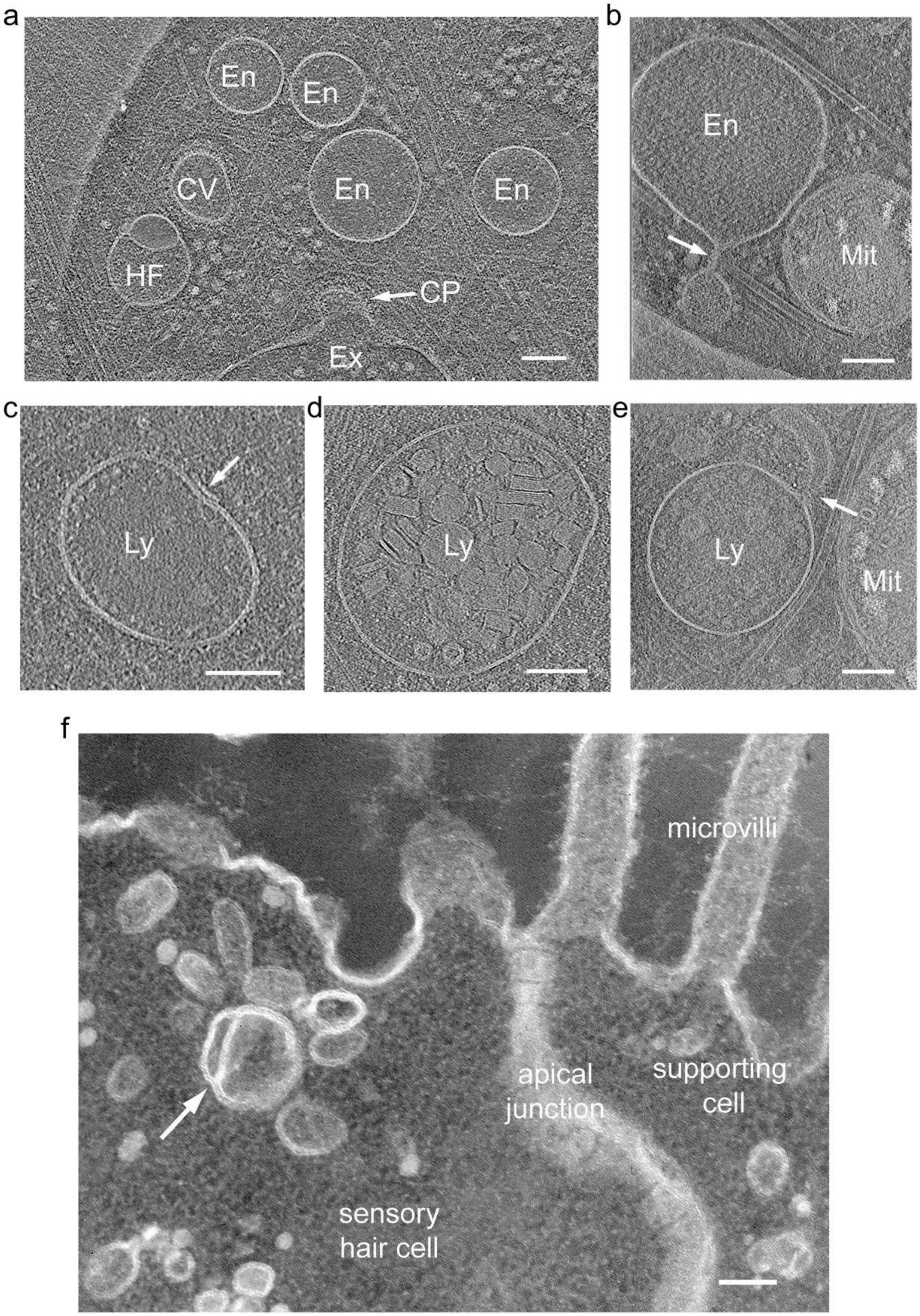

Supplementary Figure 3

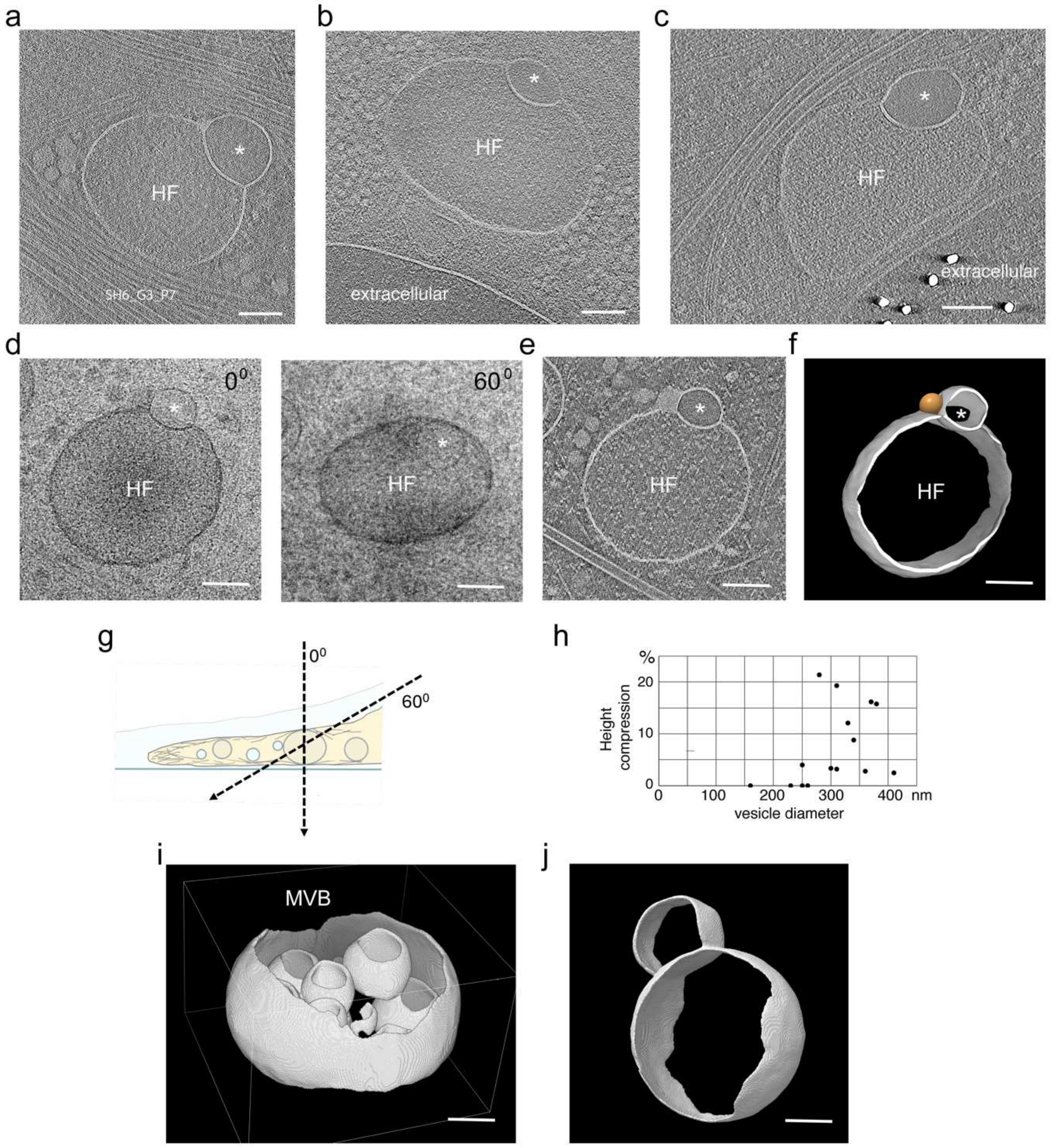

Supplementary figure 4

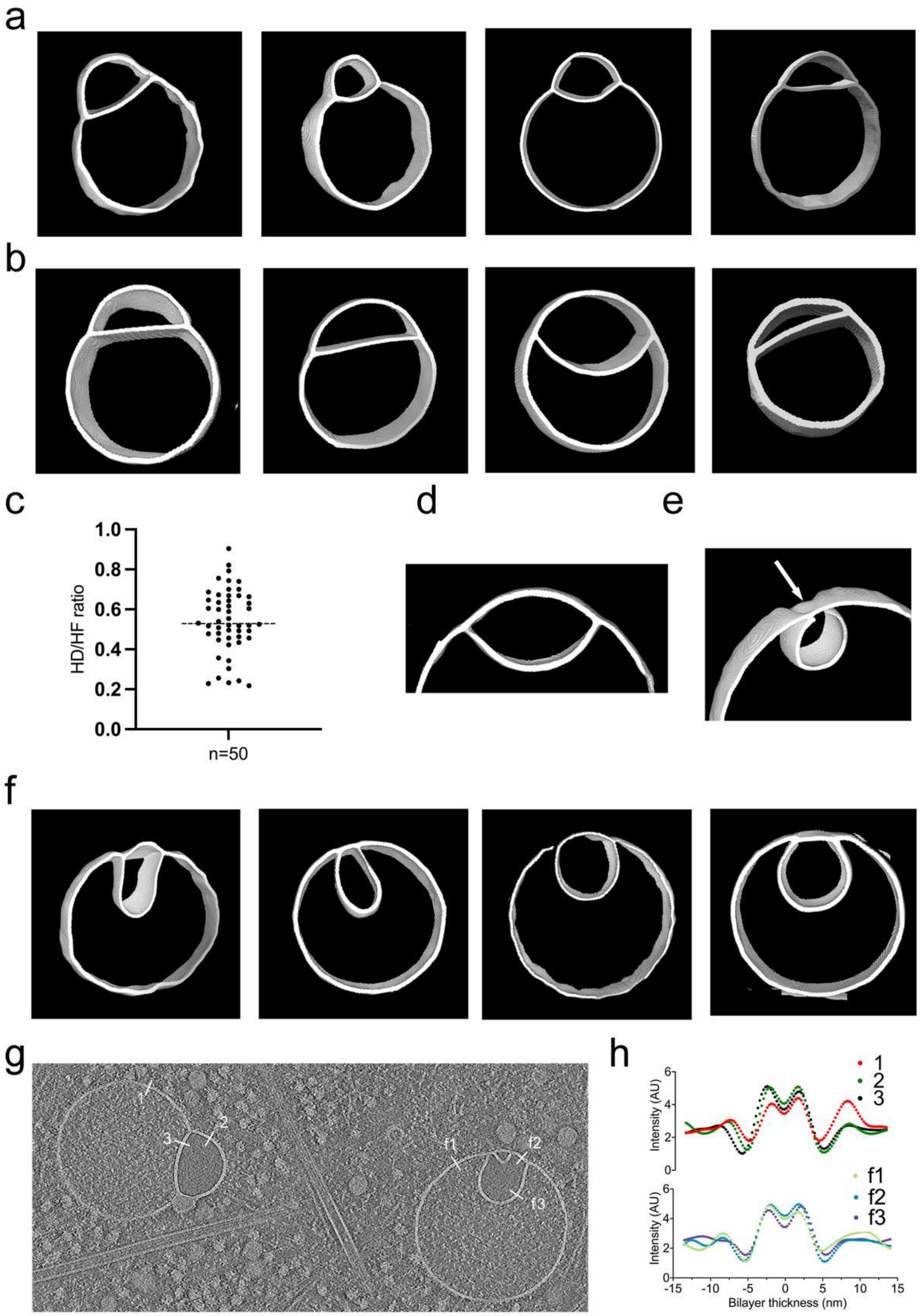

Supplementary Figure 5

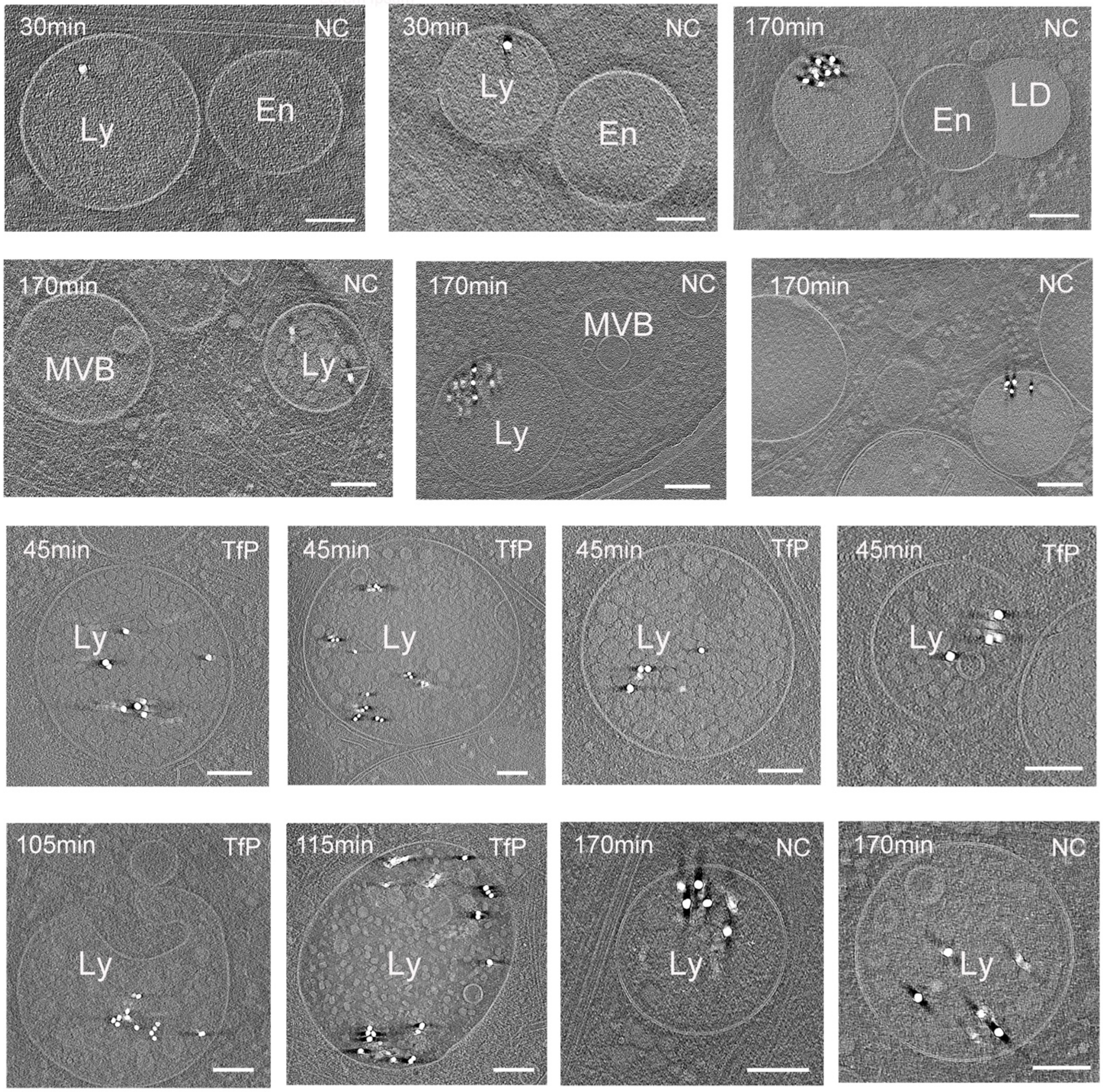

Supplementary figure 6

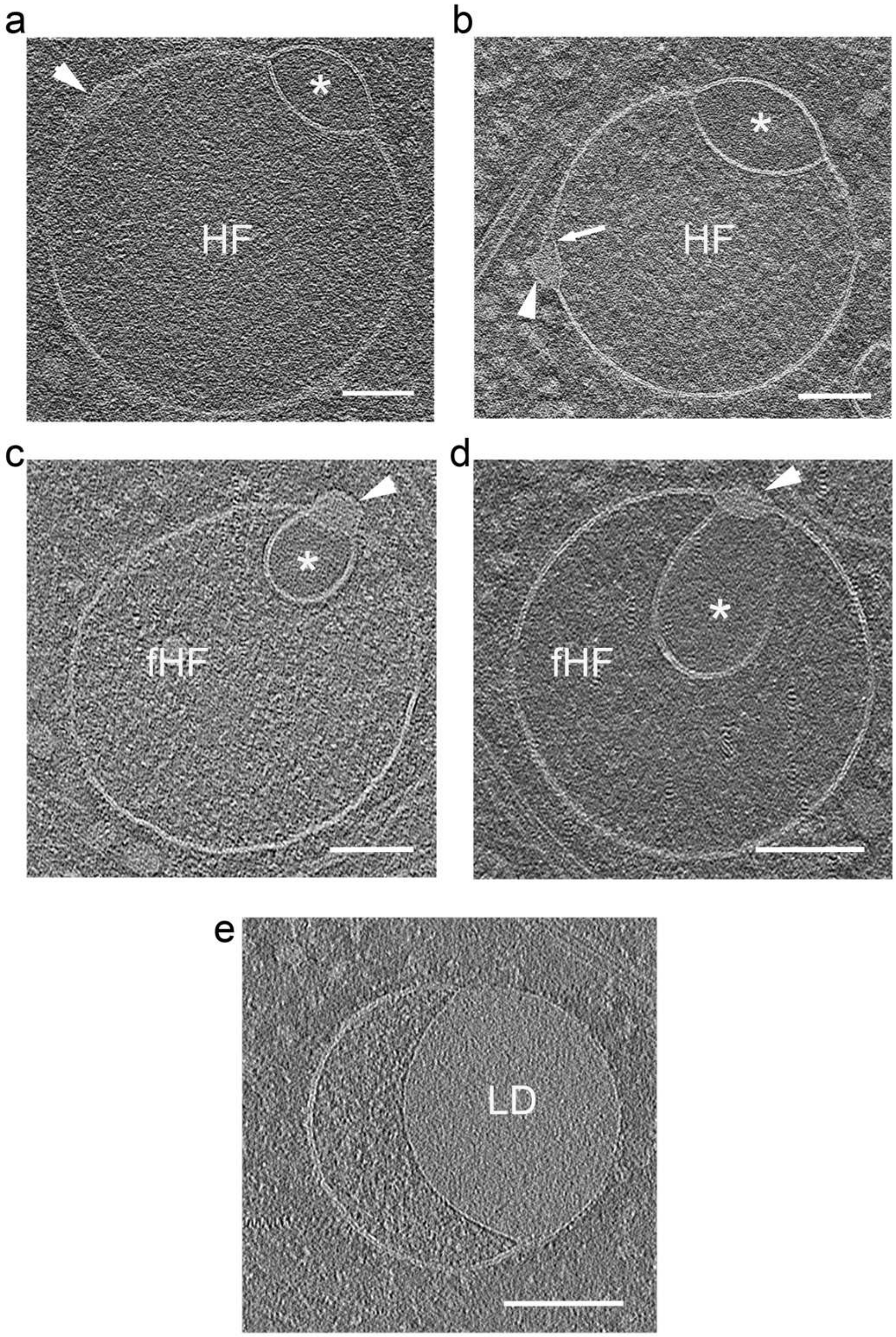

Supplementary Figure 7

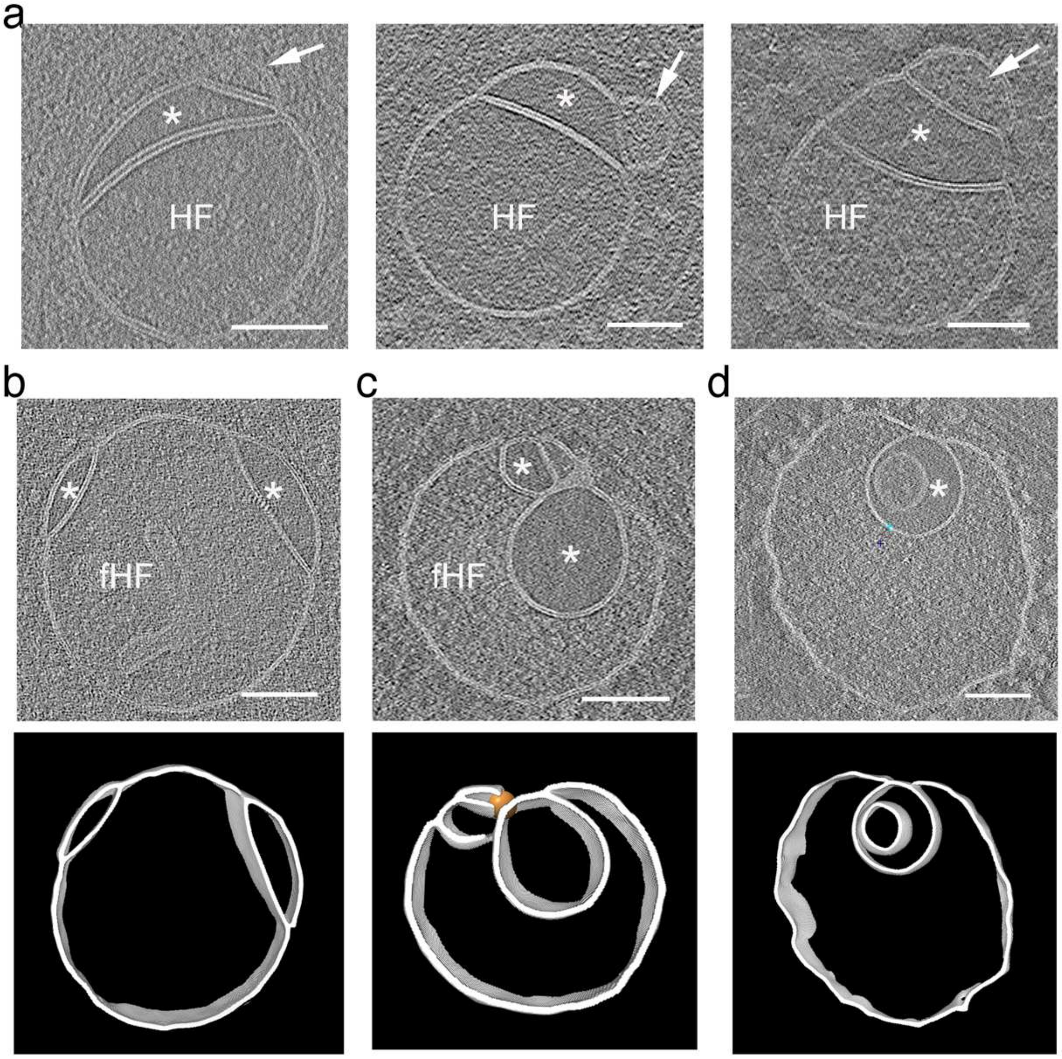

Supplementary Files

This is a list of supplementary files associated with this preprint. Click to download.

- S1.mp4
- S2.mp4
- S3.mp4
